# Transforming growth factor beta-1 (TGFB1) and peak bone mass: association between intragenic polymorphisms and quantitative ultrasound of the heel

**DOI:** 10.1186/1471-2474-6-29

**Published:** 2005-06-14

**Authors:** Peter Tzakas, Betty YL Wong, Alexander G Logan, Laurence A Rubin, David EC Cole

**Affiliations:** 1 Dept. of Laboratory Medicine and Pathobiology, University of Toronto, Toronto ON, Canada; 2 Dept. of Clinical Pathology, Sunnybrook and Women's College Health Sciences Centre, Toronto ON, Canada; 3 Prosserman Centre for Health Research, Mount Sinai Hospital, Toronto ON, Canada; 4 Dept. of Medicine, University of Toronto, Toronto ON, Canada; 5 Dept. of Rheumatology, St Michael's Hospital, Toronto ON, Canada; 6 Dept. of Paediatrics (Genetics), University of Toronto, Toronto ON, Canada

## Abstract

**Background:**

Variance of peak bone mass has a substantial genetic component, as has been shown with twin studies examining quantitative measures such as bone mineral density (BMD) and quantitative ultrasound (QUS). Evidence implicating single nucleotide polymorphisms (SNPs) of the transforming growth factor beta-1 (TGFB1) gene is steadily accumulating. However, a comprehensive look at multiple SNPs at this locus for their association with indices of peak bone mass has not been reported.

**Methods:**

A cohort of 653 healthy Caucasian females 18 to 35 years old was genotyped for seven TGFB1 SNPs. Polymorphisms were detected by restriction endonuclease digestion of amplified DNA segments.

**Results:**

The frequencies of the least common allele at G-800A, C-509T, codon 10 (L10P), codon 25 (R25P), codon 263 (T263I), C861-20T, and 713-8 delC loci were 0.07, 0.33, 0.41, 0.08, 0.04, 0.25 and 0.01, respectively. A significant association was seen between QUS Stiffness Index (QUS-SI) and the SNP at codon 10 and the linked promoter SNP, C-509T. This association remained significant after multiple regression was used to incorporate important clinical covariates – age, BMI, level of activity, family history, and caffeine intake – into the model.

**Conclusion:**

The association of QUS-SI with -509T is consistent with a gene-dose effect, while only individuals homozygous for the codon 10P allele showed a significant increase. In this cohort of young healthy Caucasian females, the T allele at position -509 is associated with greater bone mass as measured by calcaneal ultrasound.

## Background

Osteoporosis is a common disorder of aging characterized by low bone mineral density (BMD), deteriorating bone microarchitecture, and increased fracture rate. Osteoporosis and BMD are both complex traits with a strong genetic component [1,2]. Among the genes that have been associated with BMD are those encoding the vitamin D receptor [3,4], the estrogen receptors [5,6], collagen Iα1 [7], apolipoprotein E [8], and TGFB1 [9].

Abundant in bone matrix [10], secreted TGFB1 is an important regulator of osteoblast proliferation and differentiation and directly affects bone formation *in vivo *[11]. Activating mutations in humans are associated with Camurati-Engelmann disease, a disorder of progressive diaphyseal dysplasia characterized by increased BMD [12,13]. Homozygous knock-out of the TGFB1 gene in mice is associated with an osteopenic phenotype [14]. It is not surprising, therefore, that the TGFB1 locus has emerged as a strong candidate gene in the study of osteoporosis genetics.

The TGF-β1 amino acid sequence is highly conserved across mammalian species, indicating a strong selection against variant forms of the protein [15]. Variable expression or activation of TGF-β1 may therefore be associated with altered bone remodeling and different overall BMD [6]. Thus, elevated serum TGF-β1 levels are associated with osteosclerosis, and conversely, decreased serum TGF-β1 with osteopenia.

**Figure 1:**
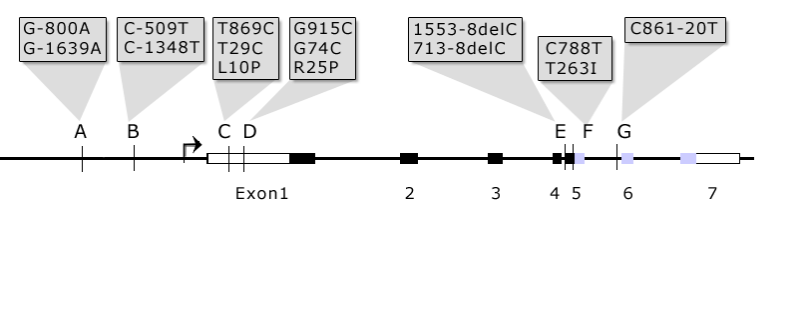
**Polymorphic loci of the TGFB1 gene. **Each locus (A to G) is shown in relation to the promoter, transcription start site (arrow), the exons (rectangles), the pre-propeptide (white rectangles), propeptide (black rectangles) and mature sequence (grey rectangles). Synonyms for each SNP (named differently by different researchers depending on annotation of position 1) are given in the shaded boxes.

Nine TGFB1 SNPs have been identified and studied. Three reside in the promoter region (C-988A, G-800A, C-509T). An insertion of a basepair is found in the 5' UTR at n.+72C. Two SNPs are in the signal sequence (L10P or n.T869C, and R25P or n.G915C), one in exon 5 (T263I or c.C788T) [16], and one in each of introns 4 and 5 (713-8delC [17] and C861-20T [18], respectively) (Figure 1).

A number of studies have examined the association of TGFB1 polymorphisms with TGF-β1 levels. In post-menopausal British women, the -509T allele was associated with higher total serum TGF-β1 [19], but this association was not confirmed in post-menopausal Chinese [20] or Japanese cohorts [21]. Similarly, the L allele at codon 10 was found to be associated with higher serum TGF-β1 levels in middle aged European women [22], but these findings were not confirmed in elderly Australian [23] or Chinese [20] women.

Other studies have also examined these allelic variants with respect to effects on bone. In 256 postmenopausal Italian women, Bertoldo et al. found strong evidence of association between the 713-8delC SNP and: (i) femoral neck BMD (FN-BMD), (ii) prevalence of hip fractures, and (iii) high bone turnover [24]. In 123 osteoporotic cases and 131 matched normal controls, Langdahl et al. found that the same 713-8delC deletion was associated with low bone mass and increased bone turnover [17]. In 76 pre-menopausal women, Keen et al. reported that the 861-20C allele was associated with higher BMD at the femoral neck, but not at the lumbar spine or with quantitative ultrasound (QUS) at the heel [18]. In a subsequent publication by Langdahl et al. involving 1168 osteoporotic cases and controls, the T allele at T861-20C was associated with significantly higher LS and FN BMD, while the T allele at C-509T was significantly associated with higher FN BMD, and the Pro allele of L10P was associated with greater FN BMD [25]. A study of the L10P variant in 625 Japanese women showed significant association of the 10P allele with higher lumbar spine (LS) BMD and total BMD [21]. This study also examined the -509 site and concluded that -509 T allele, alone or in combination with 10P, is a genetic determinant for osteoprosis. However, the more common allele at codon 10 (leucine) was found to be associated with higher LS BMD and FN BMD in middle-aged European women [22] and with higher FN BMD in Chinese elderly [20]. In 1337 elderly Australian women, the 10P allele associated with lower hip BMD and heel QUS [23]. The codon 10 polymorphism was not seen to have any association to BMD in 3382 elderly Caucasian American females [26]. In 356 healthy Japanese adolescents, however, the -509 CC homozygotes were found to have 5 to 6% greater radial BMD, and higher bone mineral content than the corresponding TT group [27].

In none of these reports were multiple SNP sites examined simultaneously in relation to peak bone mass. We therefore undertook a study of seven TGFB1 polymorphisms in relation to BMD indices as well as clinical covariates in a group of healthy young women with well characterized clinical and laboratory phenotypes to determine their relationship to peak bone mass.

## Methods

### Subjects

The 653 healthy non-related Caucasian female subjects presented in this report were recruited by advertising for a study of bone and mineral metabolism in the Greater Toronto Area, as described previously [4,6]. In this study, data on 25 women identified as originating from the Indian sub-continent were excluded.

The study protocol was approved by the Institutional Review Board of the University of Toronto (Toronto, ON) and written consent was obtained from each individual at the onset of the study [4]. Each subject completed a standardized questionnaire about lifestyle factors, menstrual and reproductive history, past history of medical diseases, current and prior medication use, history of fractures, and family history of osteoporosis. Summary clinical characteristics (mean ± SD) include: age, 27.5 ± 4.5 yr (range 18 to 35 yr); weight, 63.3 ± 11.6 kg; height, 1.65 ± 0.06 m; and BMI 23.2 ± 3.9 kg/m^2^.

### Assessment of BMD

BMD was measured at the hip (left femoral neck = FN) and lumbar spine (L2-L4 = LS) by dual-energy X-ray absorptiometry using a DPX-L absorptiometer (Lunar Corp., Madison, WI, U.S.A.). BMD was reported as grams per square centimeter. Quantitative Ultrasound (QUS) measurements were also conducted, consisting of broadband ultrasound attenuation (BUA, dB/MHz), speed of sound (SOS, m/s) and the calculated heel stiffness index (SI, % of age-matched controls), at the right heel (Lunar Achilles; Lunar Corp., Madison WI), as published previously [4]. Summary statistics for our study cohort were: LS-BMD, 1.19 ± 0.13 g/cm^2^; FN-BMD, 1.00 ± 0.12 g/cm^2^; and QUS-SI, 97.2 ± 14.1.

### DNA isolation and genotyping

DNA was extracted from EDTA-anticoagulated blood as previously described [6]. Gene fragments including the SNPs of interest were amplified by polymerase chain reaction (PCR). Of the nine SNPs known in this region, two were not included in this study. SNPs are named by either nucleotide sequence or amino acid sequence, using the most commonly reported annotation in literature. The SNP +72C was not examined, since previous work [16] revealed it to be in complete linkage with the codon 25 SNP. An additional SNP in the promoter, C-988A, was excluded because the frequency was reported to be only 0.2% [16]. The relative positions of the remaining seven intragenic SNPs are shown in Figure 1. To genotype the C861-20T SNP, previously published methods were used [18]. Methods for genotyping the other six SNPS by PCR amplification and RFLP digestion are similar to those previously published [4]. Given their pairwise proximity, three amplicons were used for genotyping: one for the pair of promoter SNPs, -800 and -509, a second for codon 10 and codon 25, and a third for 713-8delC and codon 263. Primer pairs are given in Table 1. PCR conditions were: 94°C for 1 min (denaturing), 62°C for 40 secs (primer annealing), and 72°C for 1 min + 1 second/cycle (extension) for 35 cycles. Two μl of Q-solution (Qiagen Corp) were added to the codon 10/25 PCR mixture to facilitate amplification of this GC-rich fragment. Also listed in Table 1 are the restriction endonuclease enzymes used to detect one of the two alleles (as specified). Digested DNA products were run on pre-cast Clearose BG wide-mini S-50 gels (Elchrom Scientific, Zurich, Switzerland) and stained with ethidium bromide. All genotypes were sequenced to confirm restriction digestion results.

**Table 1:** Primer sequences used for the amplification of polymorphic TGFB1 sites.

	Downstream Primer	Upstream Primer	Amplicon Size (bp)	Enzyme^a^	Cleavage^b^
-800				*Eco1*81I	Wild-type
	AGAACAGTTGGCACGGGCTT	AACGGAAGAGAGTCAGGCT	583		
-509				*Tai*I	Wild-type

Codon10				*MspA*1I	Wild-type
	CTACCTTTTGCCGGGAGACC	CACCAGCTCCATGTCGATAG	226		
Codon25	CTTTCGCCTTAGCGCCCACT	TAGTTGGTGTCCAGGGCTCG	343	*Sau*961	Wild-type

Codon 263				*Fok*1	Wild-type
				
713-8delC				*Bse*L1	Variant

^a^Restriction endonucleases (Enzyme) are shown with digestions conducted separately. ^b^Allele recognition (and cleavage) for either the major (Wild-type) or minor (Variant) allele.

### Statistical analysis

Haplotyping of -800 and -509 SNP loci was possible since both SNPs were amplified in one PCR product, and simultaneous digestion by *Eco*181 and *Tai*I produced a unique pattern of fragments for each possible haplotype [28,29]. Loci were tested for Hardy-Weinberg (HW) equilibrium of the distribution of the genotypes, and pairwise linkage disequilibrium (LD) coefficients calculated and normalized (Δ'), using methods of Weir et al. [30], and improved software as we have described [31]. Bivariate correlations between BMD, QUS-SI, and lifestyle factors, menstrual and reproductive factors, and TGFB1 genotypes were assessed using Spearman rank correlation coefficients, as reported previously [4,6]. Each TGFB1 SNP was added to the model without any clinical covariates and then with covariates used in our established model [6]. All data analyses were performed with SPSS for Windows 10.0.7 (SPSS Inc., Chicago, Illinois, U.S.A.).

## Results

### TGFB1 polymorphisms

Frequencies of the minor alleles were: codon 10 (41%), -509 (33%), C861-20T (25%), codon 25 (8%), -800 (7%), codon 263 (4%), and 713-8delC (1%). For the -800 and -509 haplotypes, 4.3% (n = 28) of the samples were doubly heterozygous, and all were in trans, as detected by sequential enzyme digestion (data not shown). Shown in Table 2 are the pair-wise standardized LD coefficients (Δ'). In this population the -509 is closely linked to codon 10 (Δ' = 0.88) and is in negative LD with 713-8delC (Δ' = -0.77). Codon 10 is also in negative LD with 713-8delC (Δ' = -0.80).

**Table 2:** TGFB1 genotype and variant allele frequencies, and pair-wise standardized LD coefficients (Δ')

				Linkage Disequilibrium Coefficients (Δ')					
				
SNP	Genotype Frequency		Variant Allele Frequency	-800	-509	Codon 10	Codon 25	713- 8DelC	Codon 263
	GG	564							
-800	GA	85	0.07						
	AA	4							

	CC	280							
-509	CT	310	0.33	-0.53***					
	TT	63							

Codon 10	TT	212							
	TC	337	0.41	-0.47***	0.88***				
	CC	97							

Codon 25	GG	555							
	GC	92	0.08	-0.66**	-0.44***	0.48***			
	CC	5							

713- 8delC	CC	626							
	C -	18	0.04	-0.61	-0.77**	-0.80***	-1.00**		
	- -	0							

Codon 263	CC	369							
	CT	242	0.01	-0.25	0.46***	0.50***	-0.32	-1.00**	
	TT	41							

C861-20T	CC	608							
	CT	43	0.25	-0.26 *	0.007	-0.10*	-0.37**	0.22*	-0.49**
	TT	2							

* P <0.05, ** P < 0.01, *** P <0.001

### Clinical characteristics by TGFB1 genotype

In comparing clinical parameters among genotype groups, no significant differences were observed for age, weight, height, calcium intake, caffeine intake, or BMI. Summary data grouped by -509 and codon 10 genotypes are shown in Table 3. Results for other genotypes are not shown, as there are no significant differences, and the other SNPs as explained below were not significant determinants of peak bone mass in this population.

**Table 3:** Clinical variables grouped by -509 and codon10 Genotype.*

	**-509**			**Codon10**		
	
	**CC ^§^(280)**	**CT (310)**	**TT (63)**	**LL (212)**	**LP (337)**	**PP (97)**
Age (yr)	27.1 ± 4.3	27.9 ± 4.7	27.5 ± 4.8	27.3 ± 4.2	27.6 ± 4.6	27.7 ± 4.9
Weight (kg)	63 ± 13	64 ± 11	62 ± 9	63 ± 2	64 ±12	62 ± 9
Height (m)	1.65 ± 0.06	1.65 ± 0.07	1.66 ± 0.07	1.65 ± 0.06	1.65 ± 0.07	1.65 ± 0.07
Calcium intake (mg/day)	590 ± 380	550 ± 340	510 ± 310	580 ± 370	570 ± 370	510 ± 300
Caffeine intake (cups/day)	1.49 ± 1.21	1.62 ± 1.61	1.33 ± 1.18	1.49 ± 1.20	1.65 ± 1.57	1.25 ± 1.21
BMI (kg/m^2^)	23 ± 3	23 ± 3	23 ± 4	23 ± 3	23 ± 3	23 ± 3

*Data expressed as mean ± SD

^§^Genotype counts (n) given in parentheses.

### TGFB1 SNPs and clinical characteristics

Correlations of QUS-SI, lumbar spine BMD, and femoral neck BMD with clinical, lifestyle and the TGFB1 genotypes are shown in Table 4. As previously reported for this cohort, LS BMD is positively correlated with age, height, weight, BMI and level of physical activity as an adolescent [4]. At the hip, significant positive correlations of BMD were found with height, weight, BMI, calcium intake, and the amount of physical activity reported at time of study and during adolescent years. Negative correlations were seen with age and the amount of caffeine consumption for the FN BMD. Heel QUS was positively correlated with the same factors as FN BMD, with the exception of calcium intake.

**Table 4:** Correlation coefficients (Spearman R) and their significance (p) are shown for QUS-SI and BMD in relation to clinical, lifestyle and TGFB1 genotypes.*

**Regression Parameters**	**Heel QUS-SI**		**Lumbar Spine BMD**		**Femoral Neck BMD**	
	
	**R**	**p**	**R**	**p**	**R**	**p**
	
						
**Clinical**						
Age	**-0.106**	**0.007**	**0.124**	**0.001**	**-0.109**	**0.005**
Height	**0.096**	**0.015**	**0.337**	**<0.001**	**0.323**	**<0.001**
Weight	**0.195**	**<0.001**	**0.166**	**<0.001**	**0.242**	**<0.001**
BMI	**0.157**	**<0.001**	**0.287**	**<0.001**	**0.208**	**<0.001**
**Lifestyle**						
Calcium Intake	0.050	0.203	0.065	0.098	**0.095**	**0.016**
Caffeine Intake	**-0.125**	**0.002**	-0.004	0.927	**-0.082**	**0.037**
Smoking	-0.027	0.499	-0.017	0.660	0.027	0.498
Currently Active	**0.104**	**0.008**	0.039	0.318	**0.083**	**0.033**
Adolescent Activity	**0.147**	**<0.001**	**0.108**	**0.006**	**0.112**	**0.004**

**SNPs**						
TGFB1 -509	**0.078**	**0.038**	0.39	0.321	0.002	0.958
TGFB1 codon 10	0.043	0.281	0.001	0.994	-0.02	0.614

*In bold are significant results (p < 0.05).

### Association of TGFB1 genotypes with BMD and QUS

There was no significant correlation of any TGFB1 SNP with BMD at FN or LS (data not shown). Of the seven loci, only the -509 SNP was significantly correlated with QUS-SI. (r = 0.078, p = 0.038). Since this SNP is in strong linkage disequilibrium with codon 10 (Δ' = 0.88) and 713-8delC (Δ' = -0.77), all three of these SNPs were included in further analysis.

**Figure 2:**
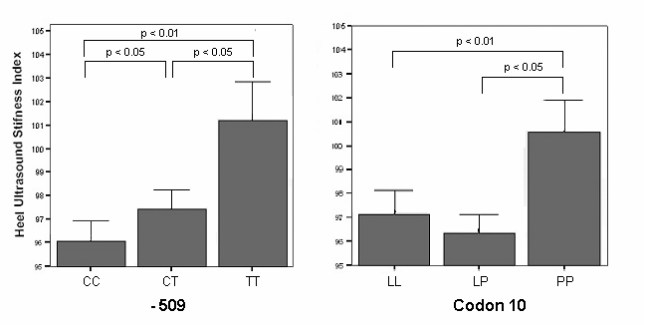
**Mean right heel ultrasound stiffness for -509 and codon10 genotypes. **Error bars represent standard error of means. P-values were obtained by Scheffé correction for multiple testing after routine ANOVA.

The number of T alleles (0, 1 or 2) at the -509 site shows significant association with QUS-SI (Figure 2). Mean QUS-SI was higher in TT homozygotes (101.2 ± 12.9) than in CT heterozygotes (97.42 ± 10.30, p = 0.04) or CC homozygotes (95.8 ± 9.31, p = 0.006) and in heterozygotes versus CC homozygotes (p < 0.05). A similar allele-dose effect was not observed with the codon10 SNP; homozygotes for the rare allele (PP group) had a significantly higher mean QUS-SI (100.6 ± 13.2) than heterozygotes (96.4 ± 14.1, p = 0.007) or LL homozygotes for the T allele (97.2 ± 14.2, p = 0.041). Adjusted QUS-SI means for the -509 SNP, after correction for age, caffeine intake and BMI (means of 27.5 years, 1.54 cups/day and 23.2 kg/m^2^, respectively), were: CC 93.6 ± 1.0, CT 95.0 ± 1.0, and TT 98.7 ± 1.9. For codon 10, adjusted means were: LL 94.8 ± 1.1, LP 93.7 ± 1.0 and PP 98.3 ± 1.5. For SNP 713-8delC, only 18 heterozygotes were found and no significant difference in QUS-SI means was observed (data not shown).

### Multivariate analysis

Based on the model previously reported by Rubin et al. [4], the seven TGFB1 genotypes were each introduced as single additive variables (variant allele counted as 0, 1 or 2) to the original multivariate analysis to test for significance. The LS BMD model included the following clinical variables: weight, physical activity at time of study, and physical activity during adolescence, age, paternal history of osteoporosis, family history of osteoporosis, and past amenorrhea greater than 3 months. None of the TGFB1 SNPs were significant predictors of LS BMD, nor were results significant for femoral neck (data not shown).

In a previously reported model from our laboratory on this group of Caucasian females the estrogen receptor (ER1) SNPs were examined [6]. ER1 *Xba*I, *Pvu*II restriction haplotypes and *AIB1 *genotypes were interactively significant in that model, which included the following clinical covariates: age, physical activity at the time of the study and physical activity in adolescent years, caffeine consumption, and BMI. When -509 and codon 10 SNPs were added as new variables to the clinical covariates model, they remained significant (Table 5). Thus, the -509 and codon 10 SNPs appear to make significant independent contributions to the explained variance of QUS-SI.

**Table 5:** Multiple linear regression analysis of genetic and clinical determinants of QUS-SI modelled to determine individual contribution of -509 and codon10.*

**-509**				**Codon 10**			
**Variables**	**F-stat**	**R^2^**	**p-value**	**Variables**	**F-stat**	**R^2^**	**p-value**

Age	6.21	**0.072**	**0.0130**	Age	5.68	**0.069**	**0.0175**
Caffeine Intake	12.74	**0.084**	**0.0004**	Caffeine Intake	9.54	**0.077**	**0.0021**
BMI	19.24	**0.185**	**<0.0001**	BMI	18.75	**0.185**	**<0.0001**
Active as adolescent	7.46	**0.107**	**0.0065**	Active as Adolescent	9.06	**0.120**	**0.0027**
Currently Active	8.51	**0.082**	**0.0037**	Currently active	7.43	**0.078**	**0.0066**
Relatives with Osteoporosis	1.98	0.008	0.1598	Relatives with Osteoporosis	1.95	0.007	0.1627
^§^-509 SNP	3.18	**0.056**	**0.0422**	^§^Codon 10	3.32	**0.062**	**0.0369**

*Shown for each dependent variable are the individual F-statistics (F-stat), the variance attributable to that variable (R^2^, based on Type III sum of squares), and the significance (p-value).

^§ ^The -509 and codon10 SNPS are significant (P <0.05) when added to the previously reported model [6]

## Discussion

TGF-β1 is a secreted factor that plays an important role in bone remodelling. It is a potent stimulator of osteoblastic bone formation, causing chemotaxis, proliferation and differentiation in committed osteoblasts [11]. Although *in vitro *experiments have led to conflicting reports about the effects of exogenous TGF-β1 in cultured osteoblast systems [32,33], secreted TGF-β1 leads to matrix growth and osteoblast stimulation *in vivo*, while it inhibits mineralization, osteoclast differentiation and the resorptive activity of mature osteoclasts [34,35]. Mouse knockouts for TGFB1 have skeletal defects, including shortened long bones and decreased tibial BMD. In humans, however, mutations found in the pro-peptide of TGFB1 are associated with Camurati-Engelman Disease (CED), an autosomal dominant disorder manifesting as periosteal and endosteal thickening of the long bone diaphyses [12,13]. Most of these mutations lie in the Latent Associated Peptide (LAP) that is cleaved from the mature TGF-β1 peptide. LAP subsequently binds to mature TGF-β1 to form an inactivated secretory complex. Presumably, mutations that interfere with binding of LAP to the mature peptide would lead to increased TGF-β1 activation, stimulating bone remodelling, net bone deposition, and resulting in a denser skeleton.

In our cohort, allele frequencies for the -800, -509, codon 10, codon 25 and codon 263 SNPs are in agreement with those published by Cambien et al. [16], and Syrris et al. [36], who examined French, Irish and UK populations. Allele frequencies in our cohort for 713-8delC and C861-20T, respectively, are comparable to those in the European Caucasians studied by Langdahl et al. and by Keen et al. [17,18]. Of the 7 SNPs examined for linkage, 713-8delC was in complete negative LD with codon 25 and codon 263. The 713-8delC SNP was in strong negative LD with -509 (Δ' = -0.77) and codon 10 (Δ' = -0.80), suggesting a single haplotype. Moreover, disequilibrium coefficients in our population were comparable to those reported by Cambien et al [16].

The two SNPs in the promoter, G-800A and C-509T, may theoretically alter the binding of RNA polymerase or other transcription factors. Luedecking et al. found that the transcriptional activity of the -509T variant allele of the TGFB1 gene was slightly greater than that of the common C allele [37]. Serum TGF-β1 concentrations are increased in a gene dose-dependent fashion, with differences in concentrations in TT homozygotes being twice that of TC individuals, when the CC genotype is taken as the referent [19]. Our results for the -509 polymorphism are consistent with this view that the T allele (high TGF-β1 producer) is associated with increased bone formation in young women, since heel QUS-SI is increased in rough proportion to the number of T alleles present. Langdahl reported a similar finding in a healthy Danish middle aged cohort, in whom higher FN BMD was associated with TT genotype [25]. In the Japanese population, however, the -509 CC genotype has been found associated with higher BMD [21]. This difference may be due to background genetic differences in the populations, ascertainment differences affecting the TGF-β1 genotype distribution in the sample set, or environmental factors. Differences in linkage disequilibrium may also contribute to this discordance, and shed light on parallel differences with codon 10 associations. In some studies, the 10Pro allele is associated with increased BMD [21,25], but not in others [20,22,23].

The TGFB1 codon 10 and 25 polymorphisms are located in the signal sequence, which is cleaved from the TGF-β1 precursor at codon 29 (Gly^29^-Leu^30 ^peptide bond). In general, signal sequence mutations affect peptide export efficiency [38]. The replacement of leucine by proline at position 10 would be expected to disrupt the alpha-helical domain, while replacement of arginine by proline at position 25 would change the characteristic polarity of the carboxyl domain of the signal sequence. In either case, altered signal peptide regulation leading to differential trafficking or export would be the likely mechanism underlying genotype-dependent differences in TGF-β1 metabolism.

The physiologic evidence for such differential expression is not extensive. Awad et al. reported a significant correlation between codon 25 genotype and amounts of TGF-β1 secreted by cultured lymphocytes stimulated *in vitro *[39]. Significant association of the codon 10 genotype with plasma TGF-β1 concentrations, BMD at lumbar spine, and vertebral fracture frequency, has been reported in controls and 2 different Japanese populations of osteoporotic patients [40]. In both control and osteoporotic groups, the association with higher plasma TGF-β1 and the 10P allele was roughly additive [9]. In a study of Japanese adolescents, those homozygous for the 10P genotype had significantly higher BMD than those homozygous for the L10 allele [27]. These results suggest that increased skeletal mineralization during puberty may be related to the presence of a 10P allele. Our study of Caucasian pre-menopausal women found no difference in BMD at either FN or LS, but found a positive association between the Leu10 allele and calcaneal QUS-SI.

Of the remaining SNPs, Langdahl et al. reported that the 713-8delC was more frequent in osteoporotic women (6/123), than controls (2/131) but no correlation to bone mass was demonstrated in the controls [25]. It is possible that linkage of this SNP with other functional sites provides an explanation for variable but genuine association with parameters of bone strength and mineral density. Indeed, we observed that the 713-8delC is in negative disequilibrium with codon 10 and -509 (Δ' = -0.77 and -0.80, respectively) in our population. Thus, previous positive results with this genotype alone may represent association with a haplotype extending across all 3 loci.

None of the TGFB1 SNPs in this study was significantly associated with either spine or hip BMD. Although apparently negative in contrast to other studies, our findings may be explained in part by the demographic characteristics of our cohort. Ours was a population of healthy young adults, whereas previous research showing an association with codon 10 and 713-8delC was based on post-menopausal women diagnosed with osteoporosis [25]. It is generally understood that studies of post-menopausal women are predominantly investigations of the rate of bone loss, whereas those in young adults are identifying genetic determinants of peak bone mass accumulation. While BMD is a measure of the density of the mineralized bone, QUS-SI is a measure of bone architecture as well [41].

The skeletal site examined for bone mass and bone quality is important. Modelling of data in twins has indicated that there are both common and specific genetic factors acting on bone at different skeletal sites and on different aspects on bone quality [42,43]. Our study suggests that TGFB1 genetic variation does not seem to be a major factor in total bone mineralization per se, at least as measured by BMD at the hip and femur.

Significant linkage disequilibrium exists between -509 and codon10 alleles and the results in our cohort (Δ' = 0.88) are no exception. However, the LD is not complete, and it is therefore no surprise that both -509 and codon 10 show association to QUS-SI, even when entered as independent variables in the multiple regression analysis. When means are compared for each genotype at a single locus, only the -509 genotypes showed a gene dosage effect, much as Yamada et al. described in a cohort of Japanese adolescents [27]. Evidence indicates that the T allele of -509 may be the more important SNP leading to an increase rate of transcription, which in turn affects bone mass. For our cohort, the multiple regression model for QUS-SI, as the dependent variable, adjusted for clinical covariates (age, height, weight, BMI, caffeine and calcium intake) and therefore these factors are likely confounders of the genotype effects seen.

Our findings must be interpreted in the context of several potential limitations. Our Caucasian cohort was recruited from an ethnically diverse urban population and admixture effects cannot be excluded. Only women were recruited, and we cannot say whether a similar association would be observed in men. Recruitment was conducted by public advertisement, and there may be significant bias toward those believing they were at higher risk for a bone related disease, particularly because of family history. However, the parameters for LS and FN BMD and for QUS-SI are comparable to other cohorts of healthy young women [4], and the associations we describe are independent of family history in our multiple regression model.

Common variations in DNA sequence are often associated with mild phenotypic effects [44]. Thus even a single SNP could account for a significant variation in bone mass so as to potentially influence subsequent fracture risk. However, the contribution of genotypes determined by all intragenic loci within a gene must be evaluated with environmental factors, in order to generate a balanced picture of gene-environment interactions for a complex quantitative trait like peak bone mass.

## Competing interests

The author(s) declare that they have no competing interests.

## Authors' contributions

PT carried out genetic tests, analysed data and drafted the manuscript.

DC conceived the study, and supervised the study design, statistical analysis, and drafting of manuscript

BW was involved in the design of the genotyping assays and sample preparation

AL was involved in study design and interpretation of the data

LR was involved in recruitment of study population, acquisition of samples, and auditing of the clinical database

All authors read and approved the final manuscript

## Pre-publication history

The pre-publication history for this paper can be accessed here:


